# Ceftazidime-Avibactam (C/A) Resistant, Meropenem Sensitive KPC-Producing *Klebsiella pneumoniae* in ICU Setting: We Are What We Are Treated with?

**DOI:** 10.3390/ijms24054767

**Published:** 2023-03-01

**Authors:** Silvia Corcione, Ilaria De Benedetto, Nour Shbaklo, Giulia Torsello, Tommaso Lupia, Gabriele Bianco, Rossana Cavallo, Luca Brazzi, Giorgia Montrucchio, Francesco Giuseppe De Rosa

**Affiliations:** 1 Department of Medical Sciences, Infectious Diseases, University of Turin, 10124 Turin, Italy; 2 School of Medicine, Tufts University School of Medicine, Boston, MA 02111, USA; 3 Department of Anesthesia, Intensive Care and Emergency—‘Città della Salute e della Scienza’ Hospital, 10126 Turin, Italy; 4 Infectious Diseases Unit, Cardinal Massaia Hospital, 14100 Asti, Italy; 5 Microbiology and Virology Unit, University Hospital Città della Salute e della Scienza di Torino, 10126 Turin, Italy

**Keywords:** KPC, carbapenemases, ceftazidime/avibactam, resistance, D179Y

## Abstract

The continuous spread of carbapenem-resistant *Klebsiella pneumoniae* (CP-Kp) strains presents a severe challenge to the healthcare system due to limited therapeutic options and high mortality. Since its availability, ceftazidime/avibactam (C/A) has become a first-line option against KPC-Kp, but C/A-resistant strains have been reported increasingly, especially with pneumonia or prior suboptimal blood exposure to C/A treatment. A retrospective, observational study was conducted with all patients admitted to the Intensive Care Unit (ICU) dedicated to COVID-19 patients at the City of Health & Sciences in Turin, between 1 May 2021 and 31 January 2022, with the primary endpoint to study strains with resistance to C/A, and secondly to describe the characteristics of this population, with or without previous exposure to C/A. Seventeen patients with colonization or invasive infection due to *Klebsiella pneumoniae*, C/A resistance, and susceptibility to meropenem (MIC = 2 µg/L) were included; the *bla*_KPC_ genotype was detected in all isolates revealing D179Y mutation in the *bla*_KPC-2_ (*bla*_KPC-33_) gene. Cluster analysis showed that 16 out of the 17 C/A-resistant KPC-Kp isolates belonged to a single clone. Thirteen strains (76.5%) were isolated in a 60-day period. Only some patients had a previous infection with non-mutant KPC at other sites (5; 29.4%). Eight patients (47.1%) underwent previous large-spectrum antibiotic treatment, and four patients (23.5%) had prior treatment with C/A. The secondary spread of the D179Y mutation in the *bla*_KPC-2_ during the COVID-19 pandemic needs to be addressed constantly by an interdisciplinary interaction between microbiologists, infection control personnel, clinicians, and infectious diseases consultants to properly diagnose and treat patients.

## 1. Introductions

The continuous spread of carbapenemase-producing *Klebsiella pneumoniae* (CP-Kp) strains presents a severe challenge to healthcare systems because of the limited therapeutic options, high mortality, and allocated costs worldwide [1,2]. In the early 2000s, CP-Kp was first identified in Europe, and its prevalence has since increased [3]. Current European epidemiology ranges from sporadic imported cases and hospital outbreaks to interregional spread and endemic CP-Kp in healthcare settings [4]. Among carbapenemase producers, *Enterobacterales*, KPC-producing *K. pneumoniae* (KPC-Kp) is the most common in Italy [5,6,7,8,9]. Other carbapenemases, namely metallo beta-lactamase (MBL), New Delhi metallo beta-lactamase (NDM), and Verona integron-encoded metallo-β-lactamase (VIM), have also been found in recent years [10,11]. Mobile genetic elements (MGEs), including plasmids, transposable elements, and integrons, are perhaps the most vital factors associated with spreading carbapenemase genes among various bacterial species [12]. The global dissemination of KPC-Kp has been linked to the successful spread of a specific genetic line, designated clonal group 258 (CG258). The KPC-coding gene, *bla*_KPC_, is generally found within a Tn*4401* transposon, a mobile genetic element originating from the Tn3 transposon family which aids its dissemination [13,14].

Ceftazidime/avibactam (C/A) is a new β-lactam/β-lactamase inhibitor combination that has been available since 2015, and in Italy, since February 2018. Interestingly, avibactam is structurally distinct from other β-lactamase inhibitors used in clinical practice as it lacks a β-lactam core [15]. The mechanism of “reversible” enzymatic inhibition involves the opening of the avibactam ring, which does not include a hydrolysis but consists only in a deacylation [16]. Increasingly over recent years, C/A has become a first-line option against KPC-producing *Enterobacterales*, especially in critically ill patients with high INCREMENT-CPE scores within endemic settings, and it has been associated with significantly reduced mortality [17]. Unfortunately, episodes of colonization or infection due to C/A-resistant strains have also been reported in the literature with or without prior C/A treatment in primary and secondary resistance features [18,19,20,21,22,23,24,25,26,27,28,29,30,31,32,33,34,35,36,37,38,39,40]. Resistance to C/A is typically caused by the presence of MBLs, as avibactam does not restore their activity. Other mechanisms are the increased expression of mutated *bla*_KPC_ or other β-lactamase genes, resulting in increased copy number; modification in cell permeability, such as loss of porins; and overexpression of efflux pumps or pyruvate dehydrogenase complex enzyme variants [18,19].

Previous studies showed that the development of resistance often emerged during prolonged C/A treatment [18,19,20,21,22,23,24,25,26,27,28,29,30,31,32,33,34,35,36,37,38,39,40]. In addition, comorbidities, pneumonia, inappropriate antimicrobial plasma concentrations, critical illnesses, and exposure to other colonized patients due to infection control issues may represent other risk factors for resistance [21,22,23,24,25,26]. To note, data regarding optimal dosing regimens for C/A in critically ill patients are scarce, but emerging real-life data suggest that more aggressive pharmacokinetic/pharmacodynamic (PK/PD) targets might improve clinical efficacy and suppress resistance emergence [24,25,26]. As an example, Gaibani et al. [26] described how the development of resistance due to mutations in the *bla*_KPC_ gene in KPC-Kp strains during C/A treatment was due to initial suboptimal blood exposure to the antimicrobials that lead a selection of hybrid subpopulations.

In this report, we aimed to describe the clinical features, microbiological characteristics, and outcomes of critically ill patients admitted to the intensive care unit (ICU) with a nosocomial spread of meropenem sensitive, C/A resistant, KPC-Kp strains.

## 2. Results

**Figure 1:**
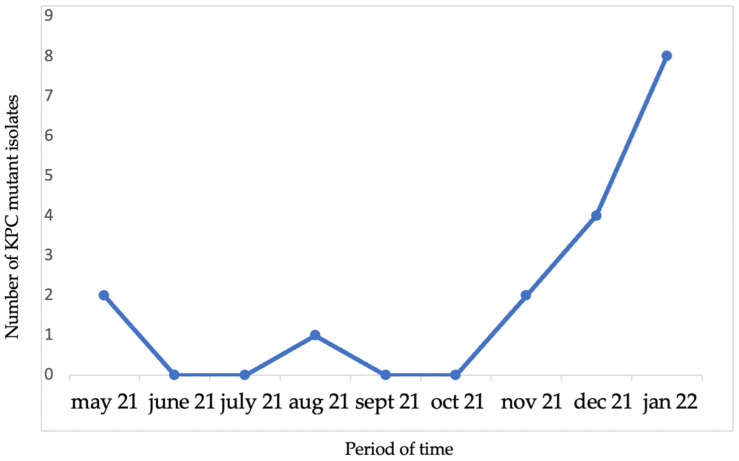
Distribution and overlap of mutant KPC-Kp isolates.

The study population consisted of 17 patients who developed a colonization or an invasive infection due to *K. pneumoniae* resistant to C/A (MIC > 256 mg/L). The 17 C/A-resistant *K. pneumoniae* isolates had the same antimicrobial susceptibility pattern (Table 1), were carbapenem susceptible (meropenem MIC 2 mg/L, imipenem ≤ 1 mg/L, ertapenem 1 mg/L), expressing an ESBL phenotype. All phenotypic carbapenemase detection methods tested negative and *bla*_KPC_ was detected in all isolates by Xpert^®^ Carba-R. Sequencing of the *bla*_KPC_ gene revealed D179Y mutation in the *bla*_KPC-2_ (*bla*_KPC-33_). Cluster analysis using Fourier-transform infrared spectroscopy showed that 16 out of the 17 C/A-resistant KPC-Kp isolates belonged to a single outbreak clone [27]. Most of the isolates overlap in the same period of admission; thirteen patients (76.4%) developed a colonization or infection in the specific time frame between December 2021 to January 2022 (Figure 1).

Half of the patients were male (9; 52.9%) and the median age was 57 years (IQR 47.5–70). Most of the patients were admitted for acute respiratory distress syndrome (ARDS) in COVID-19 (41.1%) or SARS-CoV-2 pneumonia (23.5%) (Table 2). Most cases of mutant KPC-Kp were isolated in rectal swabs (15; 88.2%) or from the respiratory tract (5; 29.4%) (Table 2). Five patients were also previously infected by C/A susceptible KPC-producing *K. pneumoniae* at other sites (29.4%). The median time from admission to C/A-resistant KPC-producing *K. pneumoniae* detection was 13 days (IQR 7–34). In addition, co-infection or concomitant superinfection was not uncommon and mostly due to bacteria (i.e., *A. baumannii*, 17.6%), viruses (i.e., SARS-CoV-2, 64.7%), or fungi (i.e., *Aspergillus*, 17.6%). Overall, 10 patients (58.8%) died during hospital admission.

Eight patients (47.0%) underwent large-spectrum antibiotic treatment before mutant-KPC-Kp isolation, and only four patients (23.5%) underwent prior treatment with C/A as detailed in Table 1. All patients previously treated with C/A had received combination therapy with fosfomycin. Following the C/A resistant isolate, 7 (41.2%) patients were treated with meropenem/vaborbactam, of which three (42.9%) were for suspected VAP.

## 3. Discussion

In this study we retrospectively reported on a cohort of seventeen critically ill patients enrolled over nine months during the COVID-19 pandemic, all of whom developed intra-hospital colonization or infection due to mutant-KPC-33 Kp with resistance to C/A, mostly without previous C/A treatment. Although relatively recently introduced, C/A use may select in vivo specific KPC-producing *Enterobacterales* resistant in patients with prolonged treatment alone or in combination [28,29,30]. Only some of these C/A-resistant strains are reported to harbor new KPC variants exhibiting single amino acid substitutions—one being Asp179Tyr (D179Y), which is particularly common and characterized by a loss of carbapenemase activity and a restoration of carbapenem susceptibility, together with a concomitant reduction of binding to avibactam [28,29]. Different types of variants encoding mutations associated with C/A resistance (e.g., KPC-41, KPC-23, KPC-14, KPC-8, and KPC-50) have been isolated in patients, with or without a history of C/A treatment [28,29,30,31,32,33,34,35,36,37,38,39,40,41,42,43]. In Italy, C/A has been available since February 2018, and the emergence of C/A-resistant *Enterobacterales* strains has been reported since 2020 [40,41,42,43].

Our data confirm that isolates harboring KPC_D179Y_ are undetectable by the main phenotypic carbapenemase detection methods, including immunochromatographic assays [37,38,42]. Since phenotypic detection methods represent the most widespread and cost-saving means of detecting carbapenemases in clinical microbiology laboratories, failure to recognize these mutated-KPC-producing isolates as alert microorganisms could easily facilitate their spread in healthcare facilities. In healthcare settings with a high circulation of KPC mutants, genotypic carbapenemase detection methods should be preferred and recommended together with antimicrobial susceptibility testing for C/A.

According to the phenotypical microbiological characteristics of our cluster of mutant-KPC-Kp, the susceptibility to carbapenems was retained; this was also reported in other case series [28,29]. Interestingly, Shields et al. [29] showed a specific *bla*_KPC_, the KPC-3 variant, as a result of plasmid transfer which determines an increase in C/A MICs and a temporary reduction in meropenem MICs ≥ 4-fold, restoring carbapenem-susceptibility in *KPC-Kp*. The microbiological data and predicted kinetic in humans suggest that infections by C/A-resistant and carbapenem-susceptible *KPC-Kp* could be treated with carbapenems. However, in a clinical setting, the effectiveness of carbapenems alone on these infections is unclear [30]. In fact, under selective pressure with carbapenems, the MICs of these compounds can increase while the species maintain their resistance to C/A [17,29], probably justifying the use of meropenem/vaborbactam whereby C/A resistance is detected and the genotypic mechanism is not determined. In particular, Shields et al. [29] showed that in vitro sublethal concentration of meropenem in patients with C/A resistant KPC-3-variant with restored susceptibility to meropenem may select for strains with high-level resistance both to C/A and meropenem. To date, no in vivo published data are available on the effectiveness of meropenem treatment in patients with such infections. On the other hand, some authors recently highlighted the in vitro [35,36] and in vivo [19,20] activity of meropenem-vaborbactam against carbapenemase-producing *Enterobacterales*, including isolates resistant to C/A.

We already reported an increased multidrug-resistant (MDR) pathogens rate during the COVID-19 period [43] and we speculate that the KPC-Kp acquisition was likely healthcare associated through cross-contamination and secondary spread. This calls for greater attention to infection control measures in addition to antimicrobial stewardship suggesting that it is not only a phenomenon of disseminated resistance or inappropriate antibiotic use, since COVID-19 patients were admitted regionally and not within the same hospital. Current infection control strategies appeared effective in containing and limiting the spread of multidrug-resistant pathogens. In clinical practice, however, it is not always simple to translate into practice regional interventions capable of having a tangible impact on the risk of health-care transmission. At the same time, control measures need to be scaled up after interventions to sustain their impact in the long term. As in many other prevention contexts, probably the most correct approach includes bundled strategies. Educational strategies for health personnel might only have an impact if combined with the correct environmental hygiene, monitoring of instruments and equipment, and safe environments and spaces. An easy-to-update data collection system, in addition to weekly meetings between microbiologists and infectious diseases specialists, can guarantee the clinical surveillance of the data and their repeated analysis over time. In addition, we highlight the importance of monitoring C/A susceptibility to prevent selection of resistance and the spread of involved genetic elements. Furthermore, a program of active surveillance for C/A-resistant strains using selective media should be implemented in the case of an outbreak sustained by C/A-resistant strains.

## 4. Materials and Methods

A retrospective observational study was conducted including all patients admitted to the regional referral ICU due to COVID-19, or COVID-19-associated reasons, at the City of Health & Science in Turin, Italy, between May 2021 and January 2022. A retrospective review of medical charts was performed to collect information about the clinical and microbiological characteristics of patients with C/A-resistant strains.

Isolates collected by various clinical specimens (rectal swabs, urine, blood, respiratory samples, etc.) were identified by MALDI-TOF/MS (Bruker Daltonics GmbH, Bremen, Germany). Rectal swabs were performed weekly as an infection control stewardship protocol at our institution. Antimicrobial susceptibility was determined by a commercially available microdilution assay (Panel NMDR, MicroScan^®^ WalkAway^®^ 96 Plus; Nyon, Beckman Coulter, Switzerland), and C/A minimum inhibitory concentrations (MICs) were confirmed by Etest (bioMérieux, Paris, France). Susceptibility data were interpreted according to current European Committee on Antimicrobial Susceptibility Testing (EUCAST) breakpoints (version 11.0, 2021; https://www.eucast.org/fileadmin/src/media/PDFs/EUCAST_files/Breakpoint_tables/v_11.0_Breakpoint_Tables.pdf, accessed on 31 December 2022). C/A-resistant Enterobacterales isolates were investigated for carbapenemase production using three phenotypical methods (Disc Diffusion Synergy test [KPC, MBL and OXA-48 Confirm Kit, Rosco Diagnostica], modified carbapenem inactivation method [mCIM], lateral flow immunoassay [NG-Test CARBA 5- NG Biotech, France], and a commercial molecular testing (Xpert^®^ Carba-R; Cepheid, Sunnyvale, CA, USA).

C/A resistant isolates were further subjected to cluster analysis using Fourier-transform infrared spectroscopy on IR Biotyper system (Bruker GmbH, Bremen, Germany) which was performed to assess the clonality and sequencing of the *bla*_KPC_ gene, as previously described [27]. The primary endpoint was to describe the features related to the appearance of strains with resistance to C/A in samples from ICU patients, and secondly to describe the clinical and microbiological characteristics of this population.

### 4.1. Statistical Analysis

Data were collected in an Excel spreadsheet and analyzed using IBM SPSS Statistics Version 28.0. Continuous variables are reported as mean (standard deviation) or median (interquartile range). Categorical variables are reported as an absolute number (percentage). Demographic and clinical characteristics of the patients were summarized through absolute frequencies and percentages for the qualitative variables, and percentiles (median, first quartile-third quartile) for the quantitative variables.

### 4.2. Ethics

The study was approved by the Local Ethical Committee (Protocol No. 000819). Data were collected in compliance with Italian laws on privacy protection. Written consent was waived according to Italian regulation, due to the retrospective and observational nature of this study in critically ill patients. Data were pooled and collected anonymously.

## 5. Conclusions

In conclusion, we report the emergence of C/A-resistant, meropenem-sensitive KPC strains in a high-endemic setting for KPC-Kp, highlighting the importance of surveillance and infection controls to stop the spreading of C/A-resistant strains. Constant interdisciplinary interaction between microbiologists, infection control personnel, clinicians, and infectious disease consultants is essential to ensure new issues are shared promptly and to implement preventive strategies that, following the COVID-19 pandemic, should be more suitable for a large regional perspective rather than single hospitals.

## Figures and Tables

**Table 1:** Susceptibility profile of mutant KPC-Kp.

Antibiotic	Sensitivity	MIC (µg/L)	MIC Breakpoint	
			S≤	R>
Ampicillin	R	>8	8	8
Amoxicillin/clavulanic acid		>32		
Piperacillin	R	>16	8	8
Pip/Tazobactam	R	>16	8	8
Ticarcillin	R	>16	8	16
Cefazolin		>16	0.001	4
Cefuroxime OV	R	>8	0.001	8
Ceftazidime	R	>32	1	4
Cefotaxime	R	16	1	2
Cefepime	R	>8	1	4
Cefixime	R	>1	1	1
Ertapenem	R	>1	0.5	0.5
Imipenem	S	≤1	2	4
Meropenem	S	2	2	8
Aztreonam	I	2	1	4
Ciprofloxacin	R	>1	0.25	0.5
Levofloxacin	R	>1	0.5	1
Amikacin	R	>16	8	8
Gentamicin	S	≤2	2	2
Tobramicin	R	>4	2	2
Colistin	R	>4	2	2
Fosfomicin IV	R	>64	32	32
Ceftazidime/avibactam	R	>8	8	8
Ceftolozane/tazobactam	R	>4	2	2
Trimetoprim-sulfametoxazole	R	>4/76	2	4
Carbapenemase		+		
Carbapenemase KPC		+		

**Table 2:** Clinical characteristics of patients with mutant KPC-Kp.

Sex/Age	Principle Diagnosis	Comorbidities	COVID-19	Time from ICU Admission	KPC Mutant Specimen	KPC Sensitive Isolation	Previous Infections	*A. baumannii* Intestinal Colonization	Previous Broad-Spectrum ATB	Previous Antifungal Therapy	CZA Treatment before Isolation	In-Hospital Mortality
F/72	ARDS COVID-19	AH, NIDDM, Obesity, malignancy	Yes	3	RS	Yes	no	no	yes	no	no	no
M/52	HAP in COVID-19		Yes	64	Blood, UC	Yes	VAP *E. coli*, MRSA, *A. baumannii*, KPC + BSI MRSE, KPC, *E. faecium*.	yes	yes	yes	yes	yes
M/56	Difficult weaning	Smoker, COPD	No	51	BAL	Yes	*Serratia marcescens* + *Pseudomonas* in BAL, CMV, BSI MRSE.	yes	yes	yes	yes	Yes
M/35	Pulmonary embolism in trauma (vv-ECMO)	Obesity	No	72	BAL	Yes	BSI *A. baumannii*, SSTI VRE	yes	yes	no	yes	no
F/61	Difficult weaning in *H. influenzae* SCAP		No	14	RS	Yes	BSI *H. influenzae*, MRSE, *S. marcescens*, *P. aeruginosa*.	no	yes	yes	no	yes
M/49	ARDS COVID-19	Obesity	Yes	15	RS	Yes	VAP *H. influenzae*	no	yes	no	no	no
F/72	ARDS COVID-19	AH, autoimmune disease	Yes	17	RS	Yes	VAP *H. influenzae*, CAPA	no	yes	Yes	no	yes
M/46	ARDS COVID-19	Previous smoker, haematological disease	Yes	10	RS	No	VAP *A. baumannii*, KPC, MSSA	yes	no	no	no	yes
M/71	Difficult weaning	AH, COPD, smoker	No	87	RS	Yes	VAP *A. baumannii*, KPC, *K. oxytoca*, *P. aeruginosa*; *C. difficile*, BSI MRSE + *Candida*	yes	yes	yes	no	yes
F/35	ARDS COVID-19, pulomonary embolism in ECMO VV	AH, NIDDM, obesity	Yes	8	RS	Yes	VAP MRSE + *K. aerogenes*	yes	yes	no	no	no
F/34	ARDS COVID-19	obesity	Yes	5	RS	Yes	VAP *E. coli* + *S. marcescens*, CAPA	no	yes	yes	no	no
F/55	ARDS COVID-19	AH, haematological disease	Yes	13	RS, blood	Yes	UTI *E. faecalis + E. coli*, BSI *S. capitis*, UTI *E. coli*	no	yes	no	no	no
F/69	ARDS COVID-19	AH	Yes	13	RS	No	VAP *P. aeruginosa*, CAPA	no	yes	yes	yes	yes
F/58	ARDS COVID-19	AH, obesity, NIDDM, respiratory and autoimmune disease	Yes	12	RS	Yes	VAP MRSA + *Proteus*	no	yes	yes	no	no
M/57	ARDS COVID-19	autoimmune disease	Yes	15	RS	Yes	VAP *P. aeruginosa*, BSI *C. albicans*, *C. auris* colonization.	no	yes	yes	no	yes
M/69	ARDS COVID-19	autoimmune disease, respiratory disease, CKD	Yes	5	RS	No	VAP *P. aeruginosa*	no	yes	no	no	yes
M/71	ARDS COVID-19	Previous smoker, NIDDM, CRF, Obesity, Malignancy, CKD	Yes	6	RS	Yes		no	yes	yes	no	yes

Abbreviations: ARDS: acute respiratory distress syndrome, AH: arterial hypertension, BAL: broncoalveolar lavage, VAP: ventilator associated pneumonia, BSI: blood-stream infection, CAPA: COVID-19 Associated Pulmonary Aspergillosis, CKD: chronic kidney disease, COPD: Chronic obstructive pulmonary disease, CRF: chronic renal failure, HAP: hospital-acquired pneumoniae, KPC: *Klebsiella pneumoniae* carbapenemase, MRSE: Methicillin-Resistant *Staphylococcus epidermidis*, MSSA: methicillin-sensitive *Staphylococcus aureus*, NIDDM: non-insulin-dependent diabetes mellitus, RS: rectal swab, SCAP: Severe Community-Acquired Pneumonia, SSTI: Skin and soft tissue infections (SSTI).

## Data Availability

Data are available under request to Authors.
